# 

**Published:** 1938

**Authors:** 


### The Medical Library of the University of Bristol

WITH WHICH IS INCORPORATED

**The Library of the Bristol Medico-Chirurgical Society.**

---

Librarian's Report for the Year ending 31st July, 1938.

The Medical Library now contains 25,814 bound volumes. During the year 282 bound volumes have been added. These include 39 from the Michell Clarke Fund, 7 from the Munro Smith Fund and 14 from the Library Grant. 56 volumes were received for review, representing a value of £39. 141 volumes were presented. 232 current periodicals are received. Other supplementary material for the Library has been purchased from the General Library Fund. 23 volumes have been withdrawn (i.e. previous edition replaced by current edition). 1,226 books have been borrowed for home reading (this includes periodicals), and 34 have been borrowed from Lewis's.

---

*The following donations have been received since the publication of the list in July,* 1938.

| | |
|---|---|
| Carnegie Institution (1) .. .. .. .. | 2 volumes. |
| Professor J. A. Nixon (2) .. .. .. | 1 volume. |
| Dr. E. Schjøtt-Rivers (3).. .. .. .. | 1 ,, |
| Professor M. W. Travers (4) .. .. .. | 1 ,, |
| Mr. E. Watson-Williams (5) .. .. .. | 1 ,, |
| Dr. P. Watson-Williams (6) .. .. .. | 15 volumes. |

Unbound periodicals have also been received from Professor E. W. Hey Groves, Professor C. Bruce Perry and Dr. J. Odery Symes.

### THE ONE HUNDRED AND SIXTY-FOURTH LIST OF BOOKS.

The figures in round brackets refer to the figures after the names of the donors. The books to which no such figures have been attached have either been bought from the Library Fund or received through the *Journal.*

**Barnhill, J. F., and Wales, E.** *Principles and Practice of Modern Otology* .. .. .. .. .. .. .. .. .. (6) 1907

**Benedict, F. G.** .. .. *Vital Energetics* .. .. .. .. .. .. (1) 1938

**Bourgeois, H., and Sourdille, M.** *War Otitis and War Deafness* (6) 1918

**Cathcart, C. W.** .. .. *The Essential Similarity of Innocent and Malignant Tumours* .. .. .. .. .. (6) 1907

**Colyer, J. F.** .. .. *Chronic General Periodontitis* .. .. (6) 1916

**Douglass, B.** .. .. *Nasal Sinus Surgery* .. .. .. .. (6) 1906

**Groves, E. W. Hey, and Brickdale, J. F.** *Textbook for Nurses* 5th Ed. (2) 1936

**Henning, G.** .. .. .. *An Historical Account of the Mineral Waters of Daviesville at Burnham, near Bridgwater* 1836

**Kerrison, P. D.** .. .. *Diseases of the Ear* .. .. .. .. .. (6) 1913

**Kyle, D. Braden** .. *Textbook of the Diseases of the Nose and Throat* .. .. .. .. .. .. 4th Ed. (6) 1907

**Lack, H. L.** .. .. *Diseases of the Nose and its Accessory Sinuses* (6) 1906

**McBride, P.** .. .. *Diseases of the Throat, Nose and Ear* .. (6) 1892

**Maxwell, J.** .. .. .. *Introduction to Diseases of the Chest* .. .. 1938

**Nagel, W.** .. .. .. *Handbuch der Physiologie des Menschen* Band 1 1905

**Parker, C. A.** .. .. *A Guide to Diseases of the Nose and Throat and their Movement* .. .. .. .. .. (6) 1906

**Paterson, D.** .. .. *Sick Children* .. .. .. .. .. 3rd Ed. 1938

**Perry, C. Bruce** .. .. *Medicine for Nurses* .. .. .. .. .. .. .. 1938

**Posey, W. C., and Wright, J. (eds.).** *A Treatise on the Diseases of the Eye, Nose, Throat and Ear.* Vol. II. (6) 1903

**Ritzman, E. G., and Benedict, F. G.** *Nutritional Physiology of the Adult Ruminant* .. .. .. .. .. .. .. (1) 1938

**Ross, Sir R.** .. .. *The Prevention of Malaria* .. .. .. (4) 1910

**Schech, P.** .. .. .. *Diseases of the Mouth, Nose and Throat* (5) 1886

**Schjøtt-Rivers, E.** .. *Hyperemesis Gravidarum* .. .. .. (3) 1938

**Short, A. Rendle, and Pratt, C. L. G.** *Synopsis of Physiology* 3rd Ed. 1938

**Thomson, W. B.** .. *Cancer—Is it Preventable?* .. .. .. (6) 1932

**Whiting, F.** .. .. *The Modern Mastoid Operation* .. .. (6) 1906

**Wright, J., and Smith, H.** *Textbook of the Diseases of the Nose and Throat* .. .. .. .. .. .. .. .. (6) 1914

TRANSACTIONS, REPORTS, JOURNALS, ETC.

Janus. First Series, Vols. 1–3. Second Series, Vols. 1–2 .. .. 1846–1853

Medical Society's Transactions. LXI. .. .. .. .. .. .. .. .. 1938

Transactions of the Ophthalmological Society of the United Kingdom. Vol. LVIII. Part 1 .. .. .. .. .. .. .. .. .. .. 1938

### Publications Received

From MESSRS. GEORGE ALLEN AND UNWIN LTD.:

*The Doctor's View of War.* Edited by H. JOULES, M.D., M.R.C.P. Price 3s. 6d.

From MESSRS. H. K. LEWIS & CO. LTD.:

*Arthritis, Fibrositis and Gout.* By CHARLES W. BUCKLEY, M.D., F.R.C.P. Price 7s. 6d.

*Psyche and the Physiologists and other Essays on Sensation.* By EDWARD GUY DRU DRURY, M.B., B.S., D.P.H. Price 5s.

*A Guide to Anatomy.* 4th Edition. By E. D. EWART. Price 12s. 6d.

*The Clinical Examination of the Nervous System.* 7th Edition. By G. H. MONRAD-KROHN, M.D., F.R.C.P. Price 8s. 6d.

*Reports on Chronic Rheumatic Diseases.* No. 4. Price 10s. 6d.

*Treatment in General Practice.* I. Articles republished from the *British Medical Journal.* 2nd Edition. Price 8s. 6d.

*Treatment in General Practice.* II. Articles republished from the *British Medical Journal.* 2nd Edition. Price 10s. 6d.

From OXFORD UNIVERSITY PRESS:

*Dysmenorrhoea: Its Ætiology, Pathology and Treatment.* By ALBERT A. DAVIS, M.D., Ch.M., M.R.C.S. Price 12s. 6d.

*Common Happenings in Childhood.* By Sir G. FREDERIC STILL. Price 5s.

From J. MILLOT SEVERN (The Brighton Phrenological and Mental Science Institution):

*Phrenology: The Language of the Mental Faculties.* By J. MILLOT SEVERN, F.P.B.S. Price 12s. 6d.

From MESSRS. JOHN WRIGHT AND SONS LTD.:

*British Journal of Surgery.* Vol. XXVI. No. 101. July, 1938. Price 12s. 6d.

### Local Medical Notes

EXAMINATION RESULTS.

**University of Bristol.**—Students of the University have recently passed the following examinations :—

M.B., Ch.B.—*Supplementary First Examination. Pass :* J. M. Allen, Margot J. Copland, J. Eskell, J. R. V. B. Gibson, J. E. G. Hope-Scott, Mildred I. Pott, T. R. Stephens.

B.D.S.—*Supplementary First Examination. Pass :* F. G. Lloyd, F. M. Warren.

L.D.S.—*Supplementary First Examination. Pass :* G. G. Davis, H. Sheffield.

**University of Cambridge.**—M.B., B.S. *Third Examination.* (*Part II*). *Pass :* C. H. Bartlett, K. G. Bergin.

**Conjoint Board.** — L.R.C.P., M.R.C.S. *Pass : In Pharmacology and Materia Medica :* E. W. J. Townsend, Megan E. Jones. *In Medicine and Surgery :* Dorothy E. M. Dickinson.* *In Medicine :* Mabel L. Glenny.* *In Pathology :* Joan E. Mackworth, A. L. T. Beddoe.

L.D.S., R.C.S. *Pass : In Anatomy and Physiology :* J. F. Williams.

**The Long Fox Memorial Lecture.**—The twenty-seventh Long Fox Memorial Lecture will be delivered at 8.30 p.m. on Tuesday, 15th November, 1938, in the Large Lecture Theatre of the H. H. Wills Physics Laboratory, Bristol University (Royal Fort) (by kind permission of the Vice-Chancellor), by Professor A. M. Tyndall, D.Sc., F.R.S. Subject : "Air as a Conductor of Electricity." The lecture will be suitable to a non-professional audience and will be illustrated by experiments.

